# Archaeogenomic analysis of Chesapeake Atlantic sturgeon illustrates shaping of its populations in recovery from severe overexploitation

**DOI:** 10.1098/rspb.2024.1145

**Published:** 2024-10-09

**Authors:** Natalia A. S. Przelomska, Matthew T. Balazik, Audrey T. Lin, Leslie A. Reeder-Myers, Torben C. Rick, Logan Kistler

**Affiliations:** ^1^ Department of Anthropology, National Museum of Natural History, Smithsonian Institution, Washington, DC 20560, USA; ^2^ School of the Environment and Life Sciences, University of Portsmouth, Portsmouth PO1 2DY, UK; ^3^ Environmental Laboratory, Engineer Research and Development Center, Vicksburg, MS 39180, USA; ^4^ Center for Environmental Studies, Virginia Commonwealth University, Richmond, VA 23284, USA; ^5^ Richard Gilder Graduate School, American Museum of Natural History, New York, NY 10024, USA; ^6^ Department of Anthropology, Temple University, Philadelphia, PA 19122, USA

**Keywords:** Atlantic sturgeon, colonial Jamestown, conservation archaeogenomics, mitochondrial genomes, seasonal spawning, zooarchaeology

## Abstract

Atlantic sturgeon (*Acipenser oxyrinchus* ssp. *oxyrinchus*) has been a food resource in North America for millennia. However, industrial-scale fishing activities following the establishment of European colonies led to multiple collapses of sturgeon stocks, driving populations such as those in the Chesapeake area close to extinction. While recent conservation efforts have been successful in restoring census numbers, little is known regarding genomic consequences of the population bottleneck. Here, we characterize its effect on present-day population structuring and genomic diversity in James River populations. To establish a pre-collapse baseline, we collected genomic data from archaeological remains from Middle Woodland Maycock’s Point (c. 200–900 CE), as well as Jamestown and Williamsburg colonial sites. Demographic analysis of recovered mitogenomes reveals a historical collapse in effective population size, also reflected in diminished present-day mitogenomic diversity and structure. We infer that James River fall- and spring-spawning populations likely took shape in recent years of population recovery, where genetic drift enhanced the degree of population structure. The mismatch of mitogenomic lineages to geographical–seasonal groupings implies that despite their homing instinct and differential adaptation manifested as season-specific behaviour, colonization of new rivers has been a key ecological strategy for Atlantic sturgeon over evolutionary timescales.

## 1. Introduction

Sturgeon are people, very much like us. You must be careful which ones you eat, and make offerings and feasts when you catch them. They’re one of our Indian foods. […] Everything must be done the right way (page 36, [1]).

During the past three hundred years, human overfishing, pollution and habitat transformation have devastated marine fisheries, including the depletion of many important species in estuaries and coastal seas [2]. While restoration and conservation in many regions have had some success, historical sources often indicate that these efforts have not restored pre-industrial levels of abundance of marine and estuarine species [2,3]. Such invaluable historical baselines can be built from historical, archaeological and paleontological records, yet remain elusive for many affected fish species. Here, we focus on endangered Atlantic sturgeon (*Acipenser oxyrinchus* ssp. *oxyrinchus*) in the Chesapeake Bay as a model for integrating archaeological and modern genetic data to understand past and present diversity and abundance, providing contextual relevance to long-term restoration and conservation of this iconic species, and with implications for other anadromous and marine fishes worldwide.

Sturgeons (family Acipenseridae) are an ancient lineage of anadromous fishes. Collectively, they are one of the most imperilled groups of fish species on the IUCN Red List of Threatened Species, with 84% of species classified as threatened [4]. Atlantic sturgeon, which inhabit estuaries and adjacent marine environments from Florida, USA, to Labrador, Canada, is a prime example of how over-harvesting can precipitate rapid and severe declines of estuarine fishes. Unsustainable Atlantic sturgeon commercial fishing caused a collapse of these fisheries in the late nineteenth century, with harvests continuing through the twentieth century, primarily for caviar production [5].

Atlantic sturgeon have a diadromous life history, characterized by long-distance migrations between freshwater and saltwater areas [6], a complex reproductive strategy and irregular spawning periodicity [7]. Their lifespan ranges from 30 to at least 60 years, with late maturation and maximum egg production at advanced ages [7]. This suite of life history characteristics was likely conducive to the persistence of Atlantic sturgeon over geological timescales [8], but in the Anthropocene these traits pose challenges to restoration of severely depleted populations. An associated consideration for conservation management is that Atlantic sturgeon’s seasonal return to natal rivers for spawning has driven genetic differentiation, implying limited gene flow between stocks. This differentiation is exemplified in the geographically delimited distinct population segments (DPSs), used for conservation management [9]. Four of these (Chesapeake, Carolina, New York Bight and South Atlantic) are listed as endangered under the Endangered Species Act (ESA) [10] and Gulf of Maine is listed as threatened [11,12]. The DPS assignments drew upon mitochondrial and microsatellite datasets that implied regional spawning populations [13,14]. More recent observations suggest that seasonal spawning runs of Atlantic sturgeon in many regions are genetically differentiated [15].

**Figure 1.**
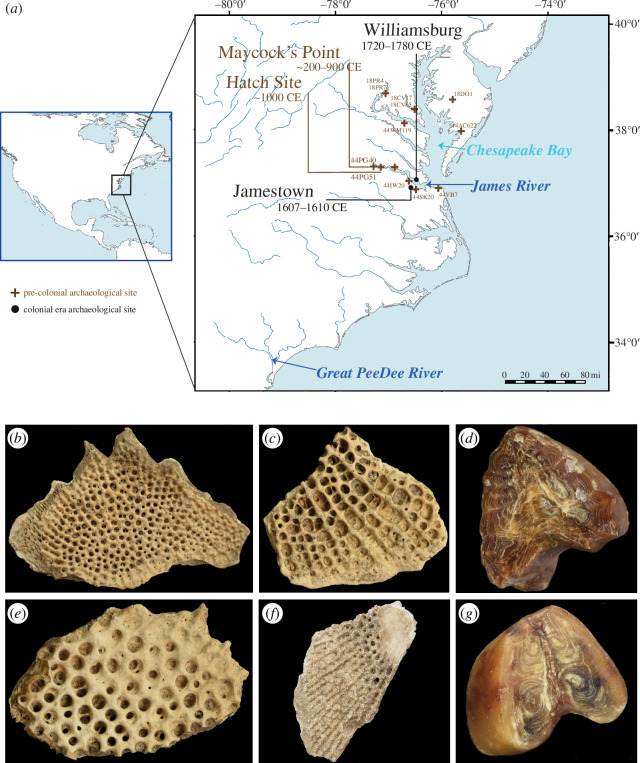
(*a*) Sampling locations for all Atlantic sturgeon used in this study, with supplementary contextual information: Chesapeake Bay archaeological sites with evidence of sturgeon. Archaeological sites marked with trinomial site IDs. Sites from which Atlantic sturgeon remains were used in this study are indicated with approximate ages of excavation, based on historical information (Jamestown), *terminus post quem* (TPQ) dates (Williamsburg) and C14-dating (Hatch Site, Maycock's Point). Rivers from which modern Atlantic sturgeon were sampled in spring and fall seasons are indicated in dark blue: James River (Chesapeake Bay DPS) and Great Pee Dee River (Carolina DPS); (*b*) scute sample 17KD-133 (Williamsburg); (*c*) scute sample 17KD-179 (Williamsburg); (*d*) spine sample JR2158H (Jamestown); (*e*) scute sample 17KD-148 (Williamsburg); (*f*) prehistoric sample H3 (Maycock’s Point); (*g*) spine sample JR2158P27 (Jamestown). Photos: Logan Kistler and Natalia Przelomska.

Because of the massive size of Atlantic sturgeon (up to 800 lbs [363 kg]) [16], their nutritious meat and roe, and their predictable seasonal migratory behaviour, these fish were an important food in pre-colonial Indigenous American societies of the mid-Atlantic Coast [17–21] (electronic supplementary material). In the Chesapeake Bay area, these include pre-colonial sites on the James River: Hatch site and Maycock’s Point (figure 1; electronic supplementary material). Radiocarbon dates from Hatch site faunal remains, which include gar, sturgeon, mussels, turtle, deer and birds, trace the material to the Late Woodland Period (c. 900–1300 CE) [22,23]. Meanwhile, radiocarbon dates available for Maycock’s Point trace human occupation to the Middle Woodland Period (c. 200–900 CE) [23]. European settlers that appeared in the seventeenth century expropriated Indigenous fisheries. The first English colony in the Americas, Jamestown, was established in 1607 and almost failed in its first few years, owing to extreme drought (evidenced by tree ring data [24]), the colonists’ poor subsistence skills and their unrealisitic expectation of a stable trading agreement with Native communities to obtain supplies of corn, squash and beans [25]. It is possible that sturgeon fishing was a lifeline that permitted the colony to persist [26,27], although archaeology suggests that this was perhaps only moderately successful, as evidenced by largely inappropriate fishing equipment (hooks) and fish bone discovered at James Fort (1607–1624) [27].

Chesapeake Bay was one area that was most heavily targeted in later commercial sturgeon fishing, supporting the second most extensive caviar fishery on the Eastern Seaboard in the late nineteenth century [28–30]. A moratorium on harvest was eventually imposed in 1998 [31] and Atlantic sturgeon is also now protected under the ESA [11,12]. Consequently, regional conservation management plans have been put in place. These are most effective when paired with information concerning Atlantic sturgeon’s unusual life history, behaviour and population genomic characteristics [15,32]. Archaeological and archaeogenomic inference have not yet been applied to Atlantic sturgeon conservation planning, yet these could contribute a valuable multi-generational perspective to help contextualize this species’ decline and recovery [3,33,34].

In this study, we designed and carried out target sequence capture of mitogenomes and nuclear exons, followed by high throughput sequencing (HTS), using a total of 22 archaeological specimens from one pre-colonial and two historic (colonial era) archaeological sites situated along the James River in present-day Virginia (figure 1*a*). For the modern panel, we sampled 50 specimens from the James River (Chesapeake Bay DPS), and to provide broader geographical context, 20 specimens from the Great Pee Dee River (Carolina DPS). With this dataset, we aimed to better understand the collapse and recovery of James River Atlantic sturgeon from a population genetic perspective. We hypothesized a decline in genetic diversity over time and set out to (i) investigate this using our time series dataset. Based on the assumption of a population bottleneck, we additionally hypothesized marked effects of genetic drift and thus specifically (ii) tested for genetic differentiation between archaeological and modern populations and (iii) between modern seasonally spawning populations.

## 2. Results

### (a) Endogenous sturgeon DNA recovered from archaeological scute and spine samples

We recovered complete or near-complete mitogenomes for 6 out of the 20 archaeological samples sequenced (> 99% of sites covered; mean depth of 24.9 ×) and 64 of the 70 modern Atlantic sturgeon samples (electronic supplementary material, tables S6, S9). In 14 additional archaeological samples, we retrieved partial coverage of the mitochondrial genome, but we conservatively analysed only highly complete mtDNA datasets. Overall, our scute samples yielded more endogenous DNA content than spine samples (electronic supplementary material, tables S6, S9). From our targeted capture and sequencing of 800 kbp of coding regions of the nuclear genome, we recovered uniquely mapped reads with an average depth of coverage of 9.0 × in modern samples (electronic supplementary material, table S5). Using 45 603 variant positions identified in quality filtered reads from the modern Atlantic sturgeon (*n* = 58), we scanned the archaeological samples for this variant set, recovering up to 503 sites in the 14 archaeological samples. The most successfully genotyped sample was from the Williamsburg excavation (W17KD179, under a coffeehouse (electronic supplementary material, tables S3c, S9)). All ancient samples displayed damage patterns consistent with post-mortem DNA damage, confirming authenticity of these samples’ association with archaeological contexts, dating to ~300, ~400 or up to 1 800 years before present (electronic supplementary material, figures S1 and S2).

### (b) Natal river and spawning season as key drivers of present-day population structure

**Figure 2.**
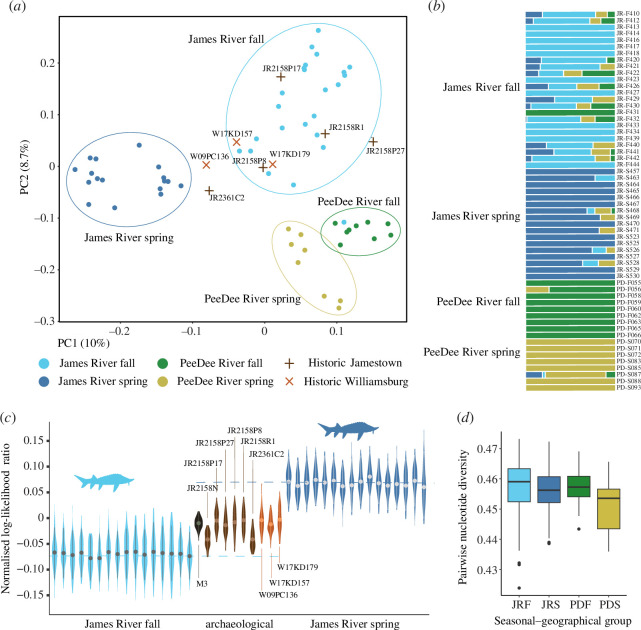
Population genomic characteristics and structure of modern Atlantic sturgeon used in this study, with the inclusion of archaeogenomic data. (*a*) Principal component analysis based on 7919 linkage disequilibrium-pruned variant sites called in modern Atlantic sturgeon, with low-coverage ancient Atlantic sturgeon samples fitted to the data structure using a Procrustes transformation, 1st and 2nd PCs plotted. (*b*) Ancestry proportions inferred for each modern Atlantic sturgeon individual, computed using maximum likelihood estimation and plotted for *K *= 4. (*c*) Probability of assignment of each James River Atlantic sturgeon individual to the modern-day spring or fall population, based on log likelihood ratios computed from pseudohaploid-called allele frequencies. Samples represented by downsampling to 200 sites x100 (modern) and x50 (ancient), where distributions for ancient samples are based on variant sets optimized for completeness in each individual sample (see electronic supplementary material for details). Dashed lines represent group means. (*d*) Relative exonic nucleotide diversity for each modern Atlantic sturgeon population. JRF, James River fall; JRS, James River spring; PDF, Pee Dee River fall; PDS, Pee Dee River spring.

We leveraged the diversity in exonic regions of modern Atlantic sturgeon to resolve contemporary population structure. Principal components analysis (PCA) based on 7919 linkage-pruned variants (figure 2*a*) shows the predicted split of samples originating from the two distinct population segments: Chesapeake Bay DPS (James River individuals) and Carolina DPS (Pee Dee River individuals). This segregation is explained by the second principal component (8.7% of genomic variance), and *F*_ST_ between James and Pee Dee Rivers was 0.0060. However, a higher degree of genetic signal (10% of variance, PC1) isolated James River spring sturgeon from the remaining distinct clusters comprising James River fall, Pee Dee River spring and Pee Dee River fall individuals. Similar results were obtained prior to considering linkage (electronic supplementary material, figure S3). *F*_ST_ between James River spring and fall was 0.0079 and between Pee Dee River spring and fall: 0.0051. Model-based clustering followed by estimation of Δ*K* suggested that 3 groups represent the uppermost level of hierarchical structure (electronic supplementary material, figures S4 and S5; here the Pee Dee individuals all cluster together). Nevertheless, we present our data according to *K* = 4, owing to the utility of the seasonal–geographical groupings (figure 2*b*). Furthermore, the number of samples with unresolved ancestry in this dataset is similar to that under *K* = 3 (electronic supplementary material, figure S5). Overall, the ancestry of James River fall individuals is the least resolved, implying admixture. In both analyses, a single James River fall sturgeon individual (JRF431) does not conform to this pattern, clustering with Pee Dee River fall genetic group. This fish is most likely a recent immigrant to the James River fall population.

### (c) Archaeological James River sturgeon do not conform to modern-day fall–spring population structuring

We predicted that the ancient samples would most likely be identifiable to the James River DPS, given their riverside archaeological site contexts. After Procrustes fitting the fragmentary datasets to the PCA (figure 2*a*), most archaeological samples showed similarity to the James River modern populations, where some projections overlap with that of the present-day fall-spawning population. Therefore, we sought to further elucidate whether the archaeological samples have a higher probability of assignment to either of the populations. Our null hypothesis was that James River sturgeon living prior to the population crash of the late nineteenth/early twentieth century will exhibit assignment to neither present-day seasonal spawning group. Following employment of a likelihood strategy based on allele frequencies of pseudohaplotype variants in modern-day populations, we found that, when grouped together, the archaeological samples cannot be confidently assigned to either population (Wilcoxon rank-sum test: *p =* 1.3 × 10^−6^ [spring], *p* = 1.7 × 10^−6^ [fall]) (electronic supplementary material, fig. S6).

Because ancient samples exhibit low genotype counts, we re-ran these analyses with downsampling of genotypes in all samples, to bootstrap our results. Furthermore, having no clear evidence for either the existence or absence of structure within the group of archaeological samples, we compared each sample individually to the respective spring and fall groups using a sample-optimized variant set in each case. Using this approach, we demonstrated that the likelihood distributions summarizing most ancient samples were still significantly different from the 50× re-sampled normalized log likelihoods of either modern population (*Z*-test, *p* < 1 × 10^−4^ in all cases; figure 2*c*, electronic supplementary material, table S7). Two Jamestown samples – JR2158N and JR2361C2 – did not display significant difference to the marginal 10% quantile of the fall population (*Z* = −0.955, *p* = 0.83 and *Z* = −0.182, *p* = 0.57, respectively) (figure 2*c*; electronic supplementary material, table S7). Thus, we demonstrate support for possible identity of a couple of ancient samples to the modern James River fall population, corroborating the general pattern suggested by the PCA. While the remainder of ancient samples show some overlap with both seasonal distributions, overall we do not find evidence that their genetic structure is consistent with the present-day differentiated fall and spring populations.

### (d) Present-day genomic diversity is higher in fall-spawning sturgeon populations

The population size reductions that endangered species’ experience often affect aspects of their genomic health, where genomic diversity is a proxy for resilience, facilitating long-term survival. Our analysis was restricted to modern Atlantic sturgeon, given the low coverage of archaeological data retrieved. The James River fall population appears to harbour higher nucleotide diversity than that of James River spring (figure 2*d*). While this difference was not statistically significant (Welch’s *t*‐test, *p* = 0.103), lower evolutionary potential of the spring population is supported by its lower effective population size ([14]; electronic supplementary material, figure S7). Our mitogenomic analysis in turn revealed statistically significant elevated genetic diversity (Watterson’s theta and π) in fall individuals compared to spring (*p <* 0.05, all comparisons). This pattern held for both river systems, with higher diversity in the Pee Dee River than the James (*p <* 0.01) (electronic supplementary material, table S8a,b).

### (e) Atlantic sturgeon mitogenomes reveal recent bottleneck and dispersed mitogenomic lineages

**Figure 3.**
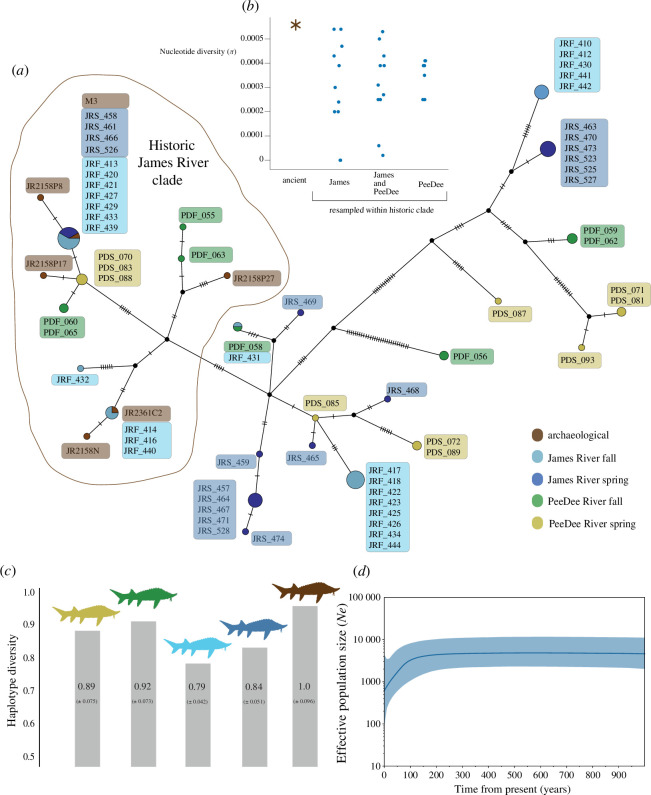
Mitogenomic structure and diversity over time based on archaeological and modern Atlantic sturgeon. (*a*) Maximum parsimony network showing relationships between Atlantic sturgeon James River and Pee Dee River mitogenomes, where 6 are archaeological (1 pre-colonial and 5 colonial era). The network shows the archaeological genomes appearing on one major branch of this network, but widely dispersed in the context of modern samples. (*b*) Nucleotide diversity (*π*) in the historic James River clade, comparing the archaeological samples to modern subsets subjected to resampling. (*c*) Haplotype diversity computed for each Atlantic sturgeon population group, using a permutation analysis. (*d*) Bayesian Skyline plot showing modelled change in effective population size (*N*_e_) over time, tracking from the present day back 3000 years, produced using BEAST, using modern mitogenomes from both James River seasonal groups and six archaeological mitogenomes.

Bayesian estimation of effective population size over time using complete mtDNA genomes suggests that there was a steady Atlantic sturgeon population in the James River prior to approximately 300–500 years ago, but from ~400 years ago, its effective population size (*N_e_*), estimated at 5300 individuals, began to diminish. Within the past ~100 years, an accelerated decline is implied, the estimated mtDNA *N_e_* dropping by approximately 71% from 3600 to 1040 in the present day (figure 3*d*). This accelerated decline in *N_e_* over the past century is in line with the known sharp declines in census size (*N_c_*) owing to stock depletion from overfishing [35]. *N*_*e*_ estimates in our sample could be affected by population structuring [36], overlapping generations [37] and variance in reproductive success and in sex ratios [38]. *N_e_* estimates can also vary depending on the genetic marker type applied and, therefore, should be taken as relative rather than absolute values. Nevertheless, they robustly demonstrate the implications of genetic diversity being lost in association with *N_c_* decline. Although mtDNA is less commonly used for recent demography, the 80% decline in *N_e_* over the past 400 years estimated here is within the range of the percentage historical *N_e_* loss estimated for 14 Atlantic sturgeon populations with a coalescent-based, migration-aware method [38].

**Figure 4.**
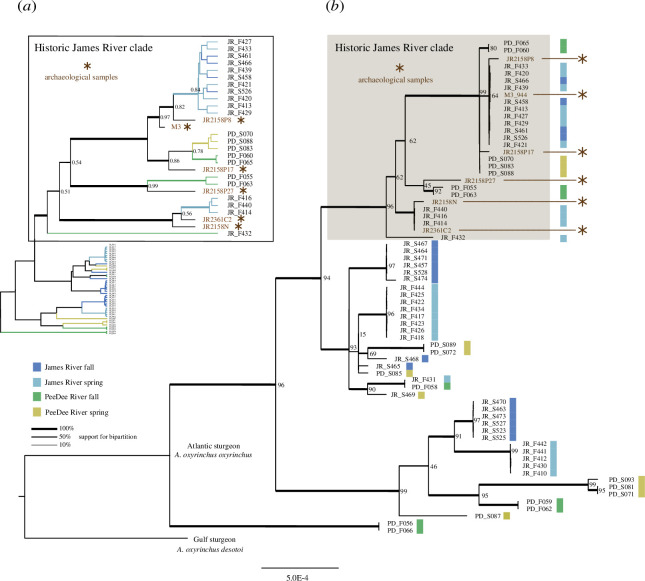
Representation of phylogenomic relationships between sequenced Atlantic sturgeon mitogenomes. (*a*) unrooted Bayesian phylogeny, with ‘James River’ clade only highlighted (see electronic supplementary material for full Bayesian phylogeny). Posterior support probabilities indicated, with thickness of branches at bipartitions corresponding to strength of support. Placements of archaeological samples within the ‘Historic James River clade’ indicated. (*b*) Maximum likelihood phylogeny, rooted with mitogenome of conspecific subspecies: Gulf sturgeon (*A. o. desotoi*). Bootstrap support indicated, with thickness of branches at bipartitions corresponding to percentage support. Placements of archaeological samples within the ‘Historic James River clade’ indicated.

Our mitogenomic phylogeny and a haplotype network reveal that the seasonal*–*geographical groupings, as defined by nuclear markers, each comprise multiple mitogenomic lineages. Present-day spring and fall James River and Pee Dee River individuals are dispersed throughout the phylogeny. Regarding the archaeological James River sturgeon, these are restricted to one large clade comprising modern (*n =* 22) and ancient (*n =* 6) samples. This clade is characterized by more unique haplotypes, as well as longer branches in the Bayesian tree (figure 4*a* and electronic supplementary material, S8), hence we refer to this as the ‘historic James River clade’ (complete posterior probability support in Bayesian phylogeny, 100% bootstrap support in maximum likelihood tree; figure 4*b*). A permutation test assessing the restriction of six archaeological mitogenomes to the identified historic clade confirms that this is unlikely due to stochastic sampling (*p* = 0.0029). Along with the higher haplotype diversity (figure 3*c*; electronic supplementary material, table S8a), this supplies evidence that mtDNA lineages were more genetically structured in the past. Furthermore, the archaeological samples have demonstrably higher mitochondrial nucleotide diversity (π) than modern samples within the historic James River clade, shown through re-sampling of the latter (one-sample *t*‐test; *p* < 0.005, when compared to all subcategories of modern data (figure 3*b*). Overall, the phylogeny is characterized by terminal polytomies (figure 4*a,b*, electronic supplementary material, figure S8). Most of these have a uniform composition of James River spring/ fall individuals and 52% of James River fall and 44% of James River spring individuals in our dataset belong to a terminal polytomy (figure 4*b*). This lends to both lower haplotype diversity among Chesapeake samples compared to South Carolina samples, as well as a significantly lower haplotype diversity for James River fall versus spring (*p* < 0.001) (figure 3*c*).

## 3. Discussion

Our genomic analyses illuminate shifts in population genomic characteristics of James River Atlantic sturgeon between the period of Indigenous human ecological management [39] and the present day. Exon-derived genomic variation defining modern-day James River Atlantic sturgeon exhibits distinct structure, which was not detected in archaeological genotypes from the same region. This structuring contributes a dimension of genetic diversity to the James River system, but simultaneously spotlights the restricted nature of these gene pools, which we deduce to have been affected by genetic drift. We detected moderate signatures of genetic diversity loss in present-day James River fishes compared to the Late Woodland and historical periods sampled using mitogenomes, which also serve to illustrate the persistence of a more diverse historical clade.

Frameworks for designating conservation and monitoring units of Atlantic sturgeon have been shaped by the spatial behavioural ecology of these anadromous fish. Atlantic sturgeon are highly philopatric [40], which has contributed to the formation of genetically distinct populations among different rivers [15]. A second behavioural driver of population structure is temporal, related to consistent spawning of individuals in either spring or fall. Our nuclear genomic variant-based PCA confirms genetic divergence between James River spring- and fall-spawning individuals, as previously inferred from microsatellite markers [41]. It is possible that this behavioural trait has been foundational to Atlantic sturgeon population dynamics over evolutionary time and in fact is a more enduring driver of differentiation than philopatry. Atlantic sturgeon populations in the southern portion of their range (Carolina and South Atlantic DPS) show instances of greater genetic similarity between groups with the same spawning season than those with different spawning seasons but natal to the same river [15]. The phenomenon of temporally reproductively isolated populations, as observed on the PCA, has an adaptive basis—it is likely the result of selection for traits conferring seasonality, e.g. responsiveness to photoperiod. Genetic control of seasonality has been evidenced in anadromous fishes such as salmon [42,43] and rainbow trout [44].

Strikingly, the genotypes we retrieved from archaeological James River samples do not conform to the present-day seasonal structure. Although historic samples yielded small variant datasets, experimental downsampling of both modern and archaeological individuals shows that individuals from highly structured populations should still be resolvable as such. Similar repercussions of a population bottleneck in terms of altering the genetic structure of terrestrial and aquatic animal populations have previously been described (e.g. [45,46]). The pattern observed here could be related to a loss of genotypes after the population crash and attendant amplification of genetic drift. Alternatively, the ancient sturgeon samples analysed could have been exclusively sampled from the James River fall populations, yet our results provide only fragmentary support for this theory. Archaeogenomic studies are most often constrained by sample availability (such as few sturgeon remains excavated at Chesapeake sites—see electronic supplementary material) as well as success of DNA extraction and endogenous content [33]. To robustly confirm our expectation from a population bottleneck*—*of substantial genotype loss triggered by very recent anthropogenic-induced depletion of sturgeon stocks*—*studies comprehensively characterizing nuclear diversity, ideally using data on the scale of whole genomes, would be required.

Humans have in fact had a protracted relationship with Atlantic sturgeon on the Eastern seaboard, dating from at least the Middle and Late Woodland Periods. During this time, the Chesapeake region was characterized by an increasing human population, cycles of social ranking and stratification, and movement and exchange between interior and coastal/estuarine regions [47]. Sturgeon played an important role as a subsistence component at some locations during this time, with the seasonal arrival of sturgeon allowing for large catches with minimum effort (electronic supplementary material, [48,49]), the archaeological record suggesting that this was often accompanied by feasting ceremonies [23,50]. Given their inferred significance, evidenced by oral histories and the presence of sturgeon remains in several archaeological assemblages [20,49,51], it is surprising that sturgeon bones, with few exceptions, are rare or absent in many Chesapeake region Middle and Late Woodland sites. This may be attributed to taphonomic bias or the specialized nature of sturgeon fishing necessitating catches away from human settlements—theories that require further investigative study (electronic supplementary material).

European colonies that were established in the early 1600s and 1700s (e.g. Jamestown and Williamsburg) are in turn associated with expropriation of sturgeon fisheries and intensified use of this estuarine resource [3,52–54]. Historical records of early colonial times refer to a robust, healthy population of sturgeon—‘there was more sturgeon here than could be devoured by dog or man*’*—quoted by Jamestown colonist John Smith [55]. While it took just a decade for colonists to find ‘meanes to take plentie of fish, as well with lines and nets’ [56], global demand for sturgeon-derived products—roe in particular—did not rise significantly until the mid-nineteenth century [5]. The short timescale of intensified use that followed was sufficient to precipitate a population crash in the early 1900s that drove Atlantic sturgeon to near-extirpation from the James River [57]. We propose that the surviving fraction of this gene pool encompassed individuals of both seasonal populations in the James River, already somewhat differentiated owing to spawning season-related adaptation. As these depauperate gene pools re-established in the James River, the strength of genetic drift was elevated owing to severely depleted population sizes. Ultimately, this led to a high level of differentiation between groups. Additional genomic work with a more exhaustive time series could probe this theory further. In this study, the Pee Dee River provides valuable context. The less marked genetic differentiation between its spawning populations could be linked to lower exploitation levels in the Carolina DPS [5] and thus a less drastic bottleneck.

The higher mitochondrial nucleotide diversity and microsatellite-based effective population size (*N_e_*) estimates for the James fall-spawning population, based on this study and [15] suggest possible scenarios of the fall population either undergoing a less severe bottleneck or historically constituting a more diverse population, as further implied by the gene flow suggested in the ADMIXTURE analysis (figure *2b*). Historical records, while sparse, do yield suggestions of a more intense harvest pressure on the spring population [3,58–60], where targeting pre-spawning females, as a source of caviar, would have had a sharp effect on population persistence. Significantly, in the eighteenth and nineteenth centuries, Atlantic sturgeon was further affected by both direct and indirect intensive fishery activities, such as those pertaining to the overlap with runs of anadromous shad (*Alosa sapidissima* & *A. mediocris*) in the spring season, which heavily impacted Atlantic sturgeon juveniles [61].

Our time-series interrogation of *N_e_* demonstrates how the studied subset of North American Atlantic sturgeon has experienced an anthropogenically accelerated decline in evolutionary potential over the past 100 years. Further, it reveals a conservation-relevant baseline of prehistoric levels of *N_e_*, in terms of orders of magnitude difference from the present day. Although conservation efforts in recent years are supporting gradual recovery in census numbers, *N_e_* estimates remain low for the James River [15,38]. This is unsurprising since the population crash was severe and the life history of Atlantic sturgeon renders its pace of both census and *N_e_* recovery times slow [62]. Furthermore, mitogenomic signal takes many generations to become observable. The mitochondrial haplotypes themselves reveal more diversity in historic James River Atlantic sturgeon compared to present-day populations, despite being mostly sampled from a single archaeological site, whereas modern-day lineages feature groups with many identical haplotypes. The diminished mitogenomic diversity is in line with genomic expectations of extinction–recolonization dynamics [46]. We suggest that, following the population collapse, re-colonization of available niche spaces with a founder effect created these pockets of mitogenomic uniformity.

Mixed maternal (mtDNA) lineages, with respect to our understanding of present-day population structuring, are observed both outside and inside of the historic James River clade. The pattern cast by this non-recombining genome reflects longer evolutionary timescales, and as such is relevant to how philopatry weighs into conservation frameworks, because it suggests pre-historical opportunistic occupation of empty niche space by Atlantic sturgeon populations beyond their natal rivers. Juvenile sturgeon thrive in an estuarine environment where the proximity of other suitable estuarine environments may increase the likelihood of re-establishing elsewhere [15]. Adults show extensive coastal movements, traversing the territory of multiple DPS [63], and their several-year hiatuses in marine environments may weaken the homing instinct [13]. Carrying capacities of different rivers utilized by Atlantic sturgeon vary widely, with estimated population sizes differing by a few orders of magnitude [8,64]. This implies that an overpopulated river might experience overspill where a proportion of the fish colonize a new river to enhance their chances of survival. Evidence for the movement of adult sturgeon into the freshwater reaches of non-natal rivers is very limited [64]. A rare example concerns a tagged James River sturgeon observed in the Patuxent River during the fall-spawning season [65]. Recolonization of Atlantic sturgeon into previously non-natal rivers has not been widely inferred (but has been suggested as an alternative explanation to long-term persistence of a small population in the Nanticoke River, a tributary of the Chesapeake Bay; [66]), possibly owing to a dearth of studies including time-depth. Recolonization becomes increasingly probable if ecological push factors are at play. In the Anthropocene, these could include rising sea and estuarine temperatures [67] and intolerable levels of salinity, pollutants and associated hypoxia, to which Atlantic sturgeon are vulnerable [68]. Future whole genome studies (WGS) in Atlantic sturgeon hold the opportunity to investigate adaptation to both their seasonal behaviour and environmental conditions, as explored in [69].

Archaeogenomic study of ichthyological remains is a growing field, which enhances our understanding of Indigenous communities of the past to whom fisheries were of crucial importance. Archaeogenomic data can confirm species identifications in cases where morphometric analyses do not provide high enough reliability [70,71], as well as provide baseline information on fish abundance, ecology, distribution and population structuring through time—an asset for informing conservation management [34,72]. The James River is listed as critical habitat for Chesapeake Bay Atlantic sturgeon [16] and has been prioritized for conservation in recent decades. In light of recent near-extirpation of Atlantic sturgeon [57], our archaeogenomic insights highlight the persistence of a pre-historic, native population and provide a window into understanding longer-term Atlantic sturgeon evolutionary dynamics. This underlines the unique value of time-depth studies for interpreting the population biology of fishes inhabiting coastal–estuarine ecosystems.

## 4. Material and methods

### (a) Sampling of zooarchaeological and present-day materials

We obtained Colonial Era sturgeon samples from archaeological collections based on excavations of two settlements on the James (Powhatan) River: Jamestown and Williamsburg. The Jamestown material came from a well and was loaned by Virginia Commonwealth University (Richmond, VA) [67]. The archaeological material comprised sturgeon spines sampled from various layers of the well (electronic supplementary material, table S3b). Larger spines (those with at least 10 growth rings) were chosen, to avoid sampling juvenile individuals that may not be native to the James River (juveniles migrate to non-natal estuaries while growing, whereas adults have very high natal return rates [4]). Williamsburg material was loaned by The Colonial Williamsburg Foundation (Williamsburg, VA) and came from six different contexts within the settlement (electronic supplementary material, table S3c). Samples from Williamsburg comprised sturgeon scutes only. Furthermore, we sourced prehistoric material previously excavated from Hatch and Maycock’s Point sites (electronic supplementary material, table S3a). These Late and Middle Woodland period sites are located further upstream of the James River, in present-day Prince George County, Virginia. We selected material morphologically identifiable as scute fragments, Atlantic sturgeon being the most likely candidate species.

We obtained a total of 50 present-day samples of Atlantic sturgeon from the James River, Chesapeake Bay Distinct Population Segment (DPS), 25 fall run individuals and 25 spring run individuals. Additionally, we sourced 20 samples from the Great Pee Dee River, Carolina DPS, 10 samples of Atlantic sturgeon from each of the fall and spring runs (electronic supplementary material, table S4). All sturgeon genetic samples were collected following requirements contained in Endangered Species Act Section 10 (a)(1)(A) research permits 20528-(01–03). Fin clip samples were stored in ethanol before extraction.

### (b) Molecular methods and initial bioinformatic data processing

We extracted DNA from all archaeological material in a clean lab at the Smithsonian Institution’s Museum Support Center following strict precautions necessary for aDNA work [73] and prepared DNA libraries following the blunt-end-single-tube method [74]. We extracted DNA from fin clips in a modern laboratory, also preparing DNA libraries according to [74]. We then conducted target bait capture using separate, custom-designed kits, respectively targeting nuclear genomic sequence and the mitochondrial genome of *A. o. oxyrinchus*. *Further details of both molecular methods and initial bioinformatic data processing are included in the electronic supplementary material.*

### (c) Inference of population genomic characteristics and structure and population assignment tests

Population structure analyses were carried out on a panel of diploid variants called in ANGSD [75]. Using PLINK v.1.9 [76], the dataset of variants called in modern samples was filtered to retain sites genotyped in at least 50% of individuals and tolerate sample missingness of up to 90%. We applied a minor allele frequency (MAF) threshold of 0.05 and pruned for linkage disequilibrium using PLINK’s *indep* function with settings: 50 5 2. The resulting 7919 variants were used for a PCA performed on the 58 Atlantic sturgeon samples that passed filtering. The eight ancient samples with the most genotyped variants were fitted to the PCA plot using a Procrustes transformation to compute the best fit of each reduced dataset to the full reference panel. For the modern samples, we computed *F*_ST_ [77] and estimated relative nuclear diversity using PLINK. We also ran ADMIXTURE v.1.3.0 [78] using as input the filtered dataset of 7919 variants with the number of hypothetical populations varying between 2 and 8, with 10 replicates with different starting seeds and employed the Δ*K* statistic to estimate the most likely number of clusters (*K*) [79], implemented in CLUMPAK [80]. S*ee electronic supplementary material for further details*.

To test whether genomic differentiation between spring and fall run James River sturgeon could have been amplified by recent population contraction, we leveraged differences in allele frequencies between the spring and fall populations and employed a likelihood-based strategy to compute an individual’s probability of assignment to a seasonal population. For this, we used the ANGSD-called pseudohaploid variants in modern James River individuals, setting a maximum missingness of genotypes at 10% and a minimum MAF of 0.1. We then used allele frequencies to define each population. Using this set of sites employed for allele frequency calculations, we explicitly tested for the probability of each ancient sample being a member of the fall- or spring-spawning groups. Based on comparison of the ancient sample’s called genotypes to the population’s frequency of corresponding genotypes in turn, we computed the sum log likelihood (lnL) of this sample belonging to a population. To account for the differences in data quantity between modern and ancient sample sets, we carried out downsampling of variants to a random selection of 200 sites, iterating each process 100 times for the main variant set and 50 times for each ancient sample-based variant set (electronic supplementary material).

### (d) Mitogenome phylogenomics and estimation of *N_e_* over time

The six archaeological samples that yielded at least 90% complete mitochondrial sequence were taken forward for analysis (electronic supplementary material, table S6, all > 99.3% complete). One came from the pre-colonial site Maycock’s Point, four from Jamestown and one from Williamsburg. We also took forward 64 modern mitogenomes genotyped to a minimum of 94% completeness. We subjected these mitochondrial consensus sequences to a multialignment, including the mitogenome of gulf sturgeon (*A. o. desotoi*) [81] as an outgroup. We used MAFFT v.7.407 [82], with the E-INS-I iterative alignment algorithm, setting a maximum of 1000 iterations. We reconstructed a maximum likelihood phylogeny using RAxML [83] with the GTR + GAMMA model and 500 bootstrap iterations.

Using the mitogenome multi-alignment, we computed Bayesian Skyline Plots using BEAST v.2.6.3 [84] to infer changes in effective population sizes of Atlantic sturgeon over time. For the prior on clock rates, we used the strict molecular clock and a lognormal distribution with a mean in real space of 1.0 × 10^–7^, upper bound of 1.0 × 10^–5^ substitutions/site/year, lower bound of 1.0 × 10^–10^ substitutions/site/year (these bounds are part of a separate uniform prior, rather than part of the lognormal distribution itself). The HKY+Γ substitution model was used, with four rate categories for gamma-distributed rates across sites. We used an exponential prior for kappa and a lognormal prior for the gamma shape prior, with default parameters for both. Because accounting for age uncertainty has negligible or minimal impacts on the resulting estimates in BEAST [85], we used the mean date estimates for all the mtDNA sequences for the analysis. Coalescent Bayesian skyline was used as the tree prior. Default settings were used for all other parameters. Posterior distributions of parameters were estimated by Markov chain Monte Carlo (MCMC) sampling. Samples were drawn every 10 000 steps over a total of at least 1 billion steps. The first 15% of samples were discarded as burn-in. We considered sampling sufficient when the effective sample size of each parameter exceeded 100. The trace files were assessed using Tracer [86] and the samples from the independent runs were merged using LogCombiner [87]. Finally, we also conducted a nesting sampling test [88] to measure the marginal likelihoods of these runs with and without the *A. o. desotoi* outgroup, confirming that leaving it out yielded a better score. Trees created with BEAST v.2.6.3 were visualized using the Maximum Clade Credibility tree type with TreeAnnotator with a 15% burnin.

### (e) Network construction and computation of mitochondrial genomic diversity

Using the mitogenome multialignment with the *A. o. desotoi* outgroup removed, we plotted all Atlantic sturgeon mitogenome haplotypes as a network, using a maximum parsimony approach in PopART [89], applying the TCS network inference method [90]. For calculation of diversity statistics for each of the population groups, we used DnaSP v.6 [91]. To test the hypothesis that nucleotide diversity in ancient sturgeon was significantly higher than in phylogenetically representative modern sturgeon, we focussed on the historic James River clade, which includes all the ancient samples (*n* = 6). Within this clade, we repeatedly (x10) sampled random sets of six modern individuals to compute diversity in DnaSP. In addition, we repeated this process with subcategories: James River only, Pee Dee River only. To further interrogate differences in diversity between seasonal–geographical groups, we used a permutation analysis with *P*-values generated from 100 re-sampling sets of combined haplotype and *π* (nucleotide) diversity frequencies using the R script Genetic_diversity_diffs [92].

## Data Availability

Raw sequencing data from the fin clip used for probe design, from nuclear target capture, and from mitochondrial target capture have been deposited in NCBI’s SRA under BioProject accession PRJNA1141358. Intermediary files and code for the analysis are available on Dryad under the following link [93]. Supplementary material is available online [94].
